# Spatiotemporal Analysis of the Spread of the COVID-19 Epidemic in Chile Using a Percolation Model

**DOI:** 10.7759/cureus.80468

**Published:** 2025-03-12

**Authors:** Mauricio Canals

**Affiliations:** 1 School of Public Health, Environmental Health Program, Universidad de Chile, Santiago, CHL

**Keywords:** chile, covid-19, pandemic, percolation, spatial epidemiology

## Abstract

Percolation describes the critical behaviour of spatial cells that progressively change their state until they compromise an entire given space. Once a threshold proportion is reached, a large continuous cell is formed that allows the space to be compromised in a continuous trajectory. This model has been used for the spatial progress of infectious disease epidemics. We propose a logistic model of space-time progression that allows an estimation of the time at which the percolation threshold is reached. In this study, we analysed the space-time progression of the COVID-19 epidemic through Chile. We first describe the process, apply the logistic model, and simulate the process on a long grid of square cells that imitates the Chilean situation. We found that, in practice, the percolation occurred when 81.63% of the communes were infected. The logistic model had an excellent fit (R^2^ = 0.967). The grid model revealed that when less than 65% of the cells were infected, no percolation events occurred. The percolation model is applicable to the spatial progression of epidemics in Chile and is an example of directed percolation. It is useful to show that at least 65% of the communes need to be infected for the entire country to be affected. Alternatively, keeping 35% of the communes free of infection would prevent the spread of an epidemic. The logistic model of the spatial spread of an epidemic allows an estimation of the time when the threshold would be reached, which constitutes a window during which mitigation or control measures can be implemented.

## Introduction

The COVID-19 pandemic affected more than 770 million people worldwide, resulting in the death of more than 7 million people. The dynamics of this disease provide evidence of the criticality of a complex system, including nonlinearity, chaotic dynamics, and sudden state changes [1,2]. The latter becomes evident at the spatial level such as the epidemic-pandemic transition.

Percolation is a physical phenomenon that describes the critical behaviour of spatial cells that progressively change their state until they compromise a whole given space. Considering a grid of spatial cells that can be in two states (i.e., infected or noninfected), it has been described as a threshold phenomenon. Above a probability pc of infection, a large cluster is formed that allows a continuous path of cells in a given state. The percolation threshold (pc) is dependent on the shape of grid cells, sites or bonds, and the existence of continuity bridges cells. This model has been used for fire spread, parasite progression in trees, and contagion between individuals with infectious diseases [3].

During the 2009 influenza A/H1N1 pandemic, a percolation model was useful to show that the pc that characterized the epidemic-pandemic transition was equal to nearly 50-60% of countries [4,5]. Several studies have been conducted on the spatial behaviour of the COVID-19 pandemic using percolation models, contributing to the study of the role of various interventions in the spread of an epidemic. For example, the role that quarantine plays in blocking the spread of COVID-19 in terms of the interaction between people and the effects it can produce on this, social distancing, and quarantines [6]. Also, Rosales et al. (2022) used this approach in Puebla, Mexico, and reported that the removal of 34% of municipalities could lead to a 63-94% decrease in the number of expected cases, thus preventing the percolation of municipalities [7].

It has been proposed that the variation in the proportion of infected countries increases over time following a logistic model that is independent of the time scale, allowing estimation of the time at which the percolation threshold would be reached [4,5]. Cuestas et al. reported that this model was an adequate fit for the 2009 influenza A/H1N1 pandemic in Argentina [8], and Maya reported its fit for the spatial progression of COVID-19 in Mexico [9].

Chile is a country on the South Pacific coast of South America. Its continental territory represents a long, narrow strip of land. Thus, its continental territory presents a challenge to the application of the epidemic percolation model since while its width is between 90 and 445 km, its length is 4270 km. This study aimed, first, to determine the usefulness of the percolation model during the spatial progression of the COVID-19 pandemic in this country, and, second, to determine the applicability of the logistic model of space-time progression and its epidemiological consequences for the mitigation and control of future epidemics.

## Materials and methods

Source of information

COVID-19 is a notifiable disease whose registry was available during the pandemic on the official government websites [10]. We used the daily records that were grouped by communes and obtained from the Ministry of Science from 2/22/2020 to 1/15/2021** **[11,12]**.**

Coding and definitions

The Chilean territory is divided into 16 administrative regions ordered from north to south. Each region is divided into smaller units called “communes”. A commune with at least one reported case was considered infected. In this case, the commune was coded with a value of 1; otherwise, it was coded with a value of 0. This is because the traceability during the pandemic was low, which meant that when a case was detected, community transmission had already occurred. Thus, a matrix with values of 1 and 0 was obtained, in which the rows represented the continental communes (343) and the columns represented the time.

Simulations (grid model)

To study what to expect from percolation in this particular geography, the phenomenon was studied theoretically using a computational model that was based on a grid of square cells derived from good results in previous studies of the progress of the A/H1N1 pandemic [4]. This model simulated the progression of infected cells in a grid of 115 rows and 3 columns (345 cells), representing approximately 343 continental communes of Chile [12]. The width of three columns was chosen considering that the median number of communes in the east to west direction in Chile is three. A percolation event was defined when there was a continuous path between infected neighbouring cells (communes) from the north to the south of Chile. Cells that shared a side or a corner were considered neighbours. For each probability p of randomly generated infection, the program studied the existence of a continuous path of infected communes, row by row, according to the positivity of infection in neighbouring communes. One hundred thousand simulations were carried out, allowing us to establish a relationship between the proportion of infected communes (= p) and the proportion of percolation events.

​​​​​​Study of the spatiotemporal progression of COVID-19 in Chile

The geographic progression of the pandemic in Chile was studied using geographic information system (GIS) maps of the reported temporal evolution of infected communes over time. The temporal progression of the proportion of infected communes as a function of time was then studied using a logistic regression model of the proportion of infected communes (*p*) as a function of the natural logarithm of time: *logit(p) = ln(p/(1-p)) = bln(t) + c*, where *b* and *c* are constants and *ln* represents the natural logarithm. This expression is the analytical solution of *dp/pdτ = r-bp*, where *dτ = dt/t* when *dp/pdτ = 0, *then *p = r/b = 1* [5].

## Results

**Figure 1:**
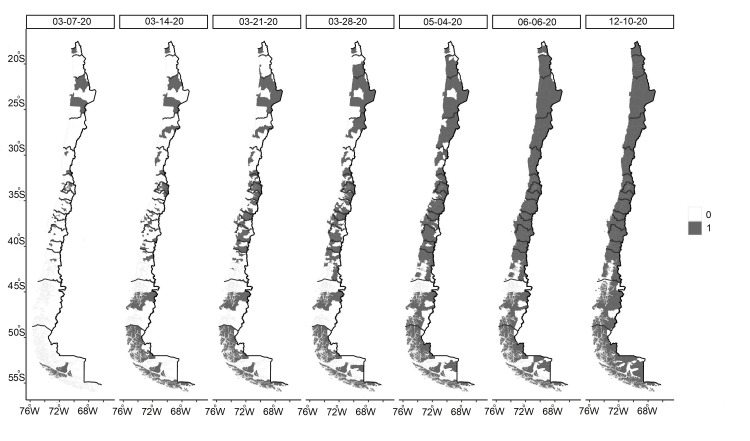
Spatial spread of the COVID-19 epidemic in Chile Spatial progression of SARS-CoV-2-infected communes in continental Chile during 2020. Infected: 1 (grey); noninfected: 0 (white) SARS-CoV-2: severe acute respiratory syndrome coronavirus 2 Figure credit: The author

**Figure 2:**
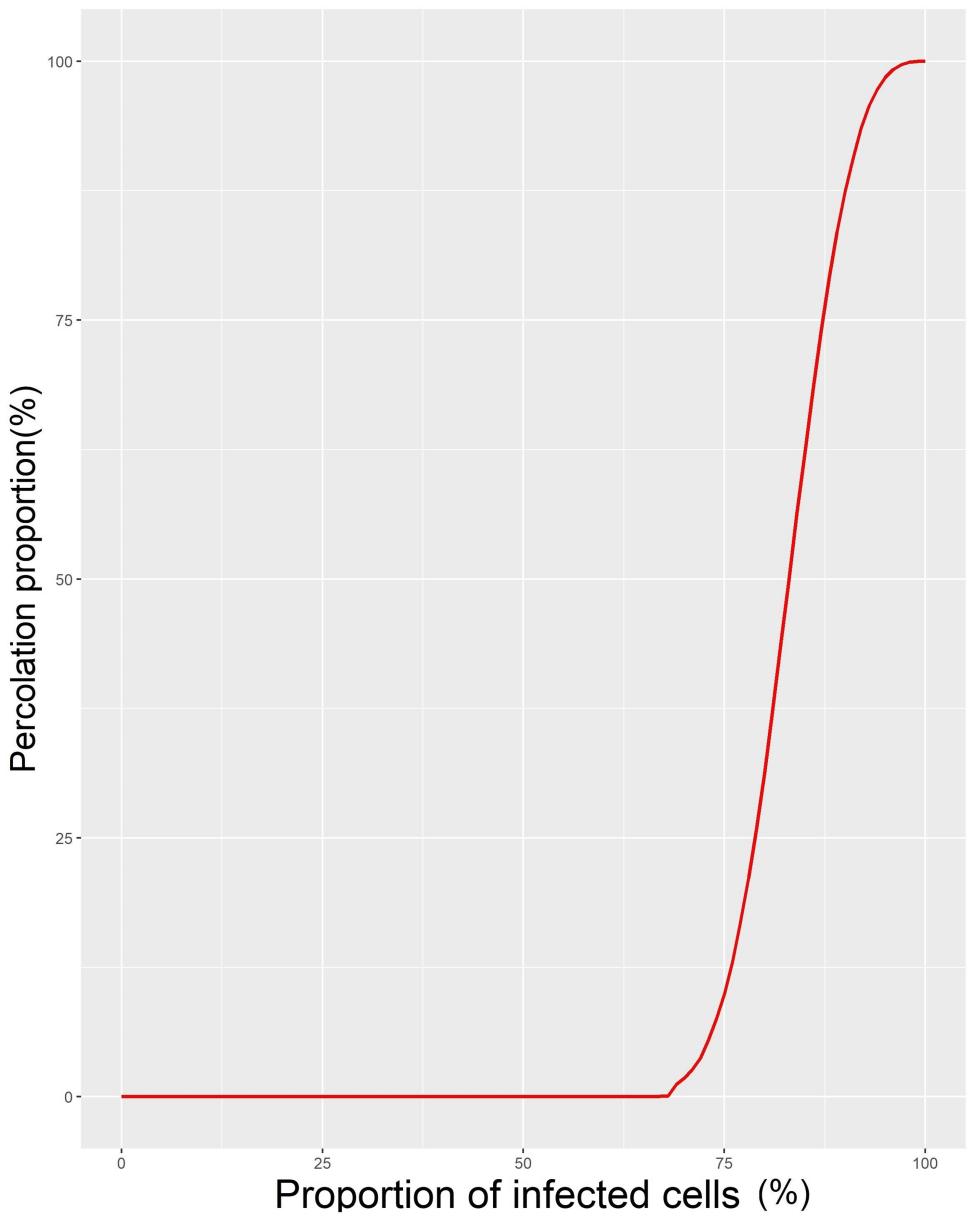
Progression of percolation events in the grid model Results of the simulation of the proportion of percolation events as a function of the proportion of randomly generated infected cells in a 3 × 115 = 345 square cell grid

In Chile, the first reported case of COVID-19 occurred in a central Chilean commune (Talca) on 03/01/2020. On 03/07/2020, there were already three foci of infection that affected i) several skipped communes in the north, ii) several communes in the centre, including the capital, Santiago, and iii) a focus in Punta Arenas, in the extreme southern region of the country. These foci increased in size, with three larger nuclei persisting as of 03/28/2020 and percolation almost obtained on 05/04/2020, except for some isolated communes in the north (Camarones, Canela, Monte Patria and Combarbalá) and far south (Cisnes, Lago Verde, Laguna Blanca, and San Gregorio), which remained noninfected until well into the pandemic. These communes are small and within the range of mobility of people. Thus, in practice, they did not interrupt the continuity of the infection process (Figure 1). The grid model revealed that with 65% of the cells infected, there were no percolation events; with 73% of the cells infected, there were only 10% of events; and with 83% of the cells infected, there were approximately 50% of events (Figure 2).

**Figure 3:**
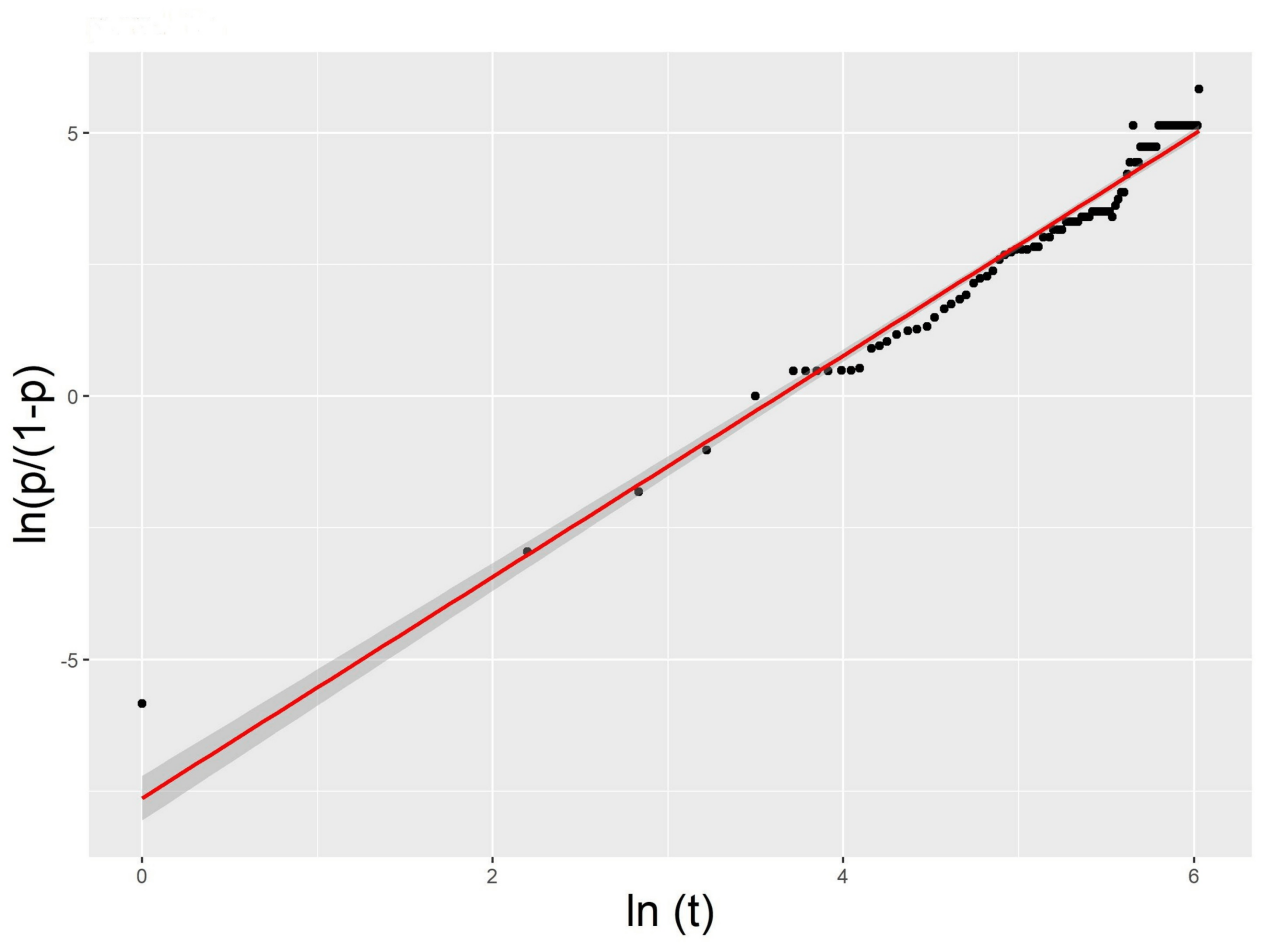
Time spread of COVID-19 in Chile Time (t) progression of the proportion of infected communes (p) in continental Chile during 2020 (points), and the logistic model fitted (red line) with its 95% confidence limits. Logit (p) = ln(p/(1-p) vs. ln(t)

The spatiotemporal progression of the pandemic in Chile closely fit the following logistic model (Table 1, Figure 3). The model obtained was: *Logit(p) = ln (p/(1-p)) = 2.137 ln(t) -7.629; F1,89 = 2589; p-value << 0.001; R2 = 0.967*.

**Table 1:** Temporal evolution of the communes infected with COVID-19 during 2020/2021 in Chile

Time (days)	Communes infected	Proportion (%)
1	1	0.3
9	17	5.0
17	48	14.0
25	91	26.5
33	172	50.1
41	212	61.8
54	213	62.1
60	216	63.0
64	244	71.1
67	248	72.3
70	253	73.8
74	262	76.4
79	266	77.6
83	268	78.1
88	271	79.0
92	280	81.6
97	288	84.0
101	292	85.1
106	296	86.3
110	299	87.2
115	307	89.5
119	310	90.4
137	321	93.6
171	327	95.3
226	333	97.1
285	341	99.4
420	343	100.0

## Discussion

The percolation model allowed us to characterize the phase change between epidemics and pandemics in COVID-19 and other infectious diseases [4,6,9]. Since its conceptualization has been described as a threshold phenomenon [3], the pc threshold is dependent on the shape of the grid cells, the existence of bonds or sites between cells, and whether percolation is directed, i.e., whether there is a dominant direction [13].

The geography of Chile allowed us to study the percolation of the COVID-19 pandemic from the north to the south. The geographic shape and organization of the communes might affect the interpretation of our results. While each square cell has eight neighbours (coordination number), the provinces in Chile have a smaller coordination number, with a median of five. Since the pc is inversely related to the coordination number [14], the percolation threshold for the communes in Chile should be somewhat higher than that in our grid model. Additionally, in the grid model, infections were generated randomly, which could explain the differences between the simulations and the actual spread observed in Chile. The model also allowed us to observe that under a probability pc = 0.65 (65% of communes), percolation events should not occur.

The observed spatiotemporal progression of the epidemic in Chile revealed three foci that progressed over time, in the north, center, and south of the country, initially showing progressive contagion to adjacent communes and later converging in a large percolating cluster, which was consistent with the grid model. In this case, the proportion of infected communes was a consequence of the state of infection in neighbouring communes. This could produce changes in the proportion necessary for the continuity of propagation. Additionally, in Chile, some communes occupy the whole width of the country; therefore, if they are not infected, they prevent the continuity of north to south infection. This situation was also reported in Mexico in isolated desert regions, where small clusters without infection were detected with values above the pc [9].

The logistic model of the spatiotemporal progression of the epidemic was an excellent fit for the progress of the epidemic. That is, the proportion of infected communes followed logistic growth until it affected all the communes (p = 1), which is in agreement with previous reports of the influenza A/H1N1 pandemic in Chile and Argentina and the COVID-19 pandemic in Mexico [4,8,9]. The time course of the epidemic could be affected by viral variants, public behavior, and mitigation efforts. Measures like lockdowns, vaccinations, and other public health efforts could slow down or change the spread of the virus, which could alter the threshold for percolation. However, apart from small fluctuations, the temporal progress curve of the proportion of infected communes does not show clear slowdowns attributable to these factors (Figure 3). Considering the threshold pc = 0.65, as proposed by our grid model, the logistic model suggested that this threshold should have been reached on Day 51 of the pandemic, with a 95% confidence interval of 36-73 days. This threshold was reached between Days 60 and 64 of the pandemic, in agreement with the model. The percolation occurred on Day 91, with almost 82% of the communes infected, where the probability of percolation estimated by the grid model was close to 50% (p = 0.438). Thus, it was very likely that this event would occur. The fact that this did not happen before was probably related to the lower coordination number of the communes than that in the grid of square cells.

Limitations

Our grid model has several limitations. First, it did not consider the geographic shapes of the communes, their boundaries, or the neighbourhood structure. However, in a previous study, we showed that square grids often adequately capture the overall behaviour of the system. For example, for the A/H1N1 pandemic, very similar percolation thresholds were obtained for squares and polygons divided into square cells, and when the geographic shape of each country was considered (0.5927, 0.5897, and 0.513, respectively) [4]. On the other hand, in the model, infections were generated randomly, which differed from what occurs during the spread of epidemics, in which contagion is more likely to occur in neighbouring communes. Future studies could consider the bridges of continuity between remote or spatially unconnected communes that can be established and facilitated through means of transport.

## Conclusions

This study suggests that the percolation model is applicable to the spatial progression of epidemics in Chile, being an example of directed percolation. From an epidemiological point of view, it was useful to show that at least 65% of the communes needed to be infected for the entire country to be affected. In contrast, randomly or deliberately keeping 35% of the communes free of infection would prevent the spread of an epidemic. Although the temporal progression of an epidemic can be affected by several factors, such as viral variants, population behavior, and various mitigation efforts, such as quarantines and vaccines, the logistic model of the spatial spread of an epidemic was an excellent fit for the actual situation, allowing an estimation of the time when the pc would be reached, which constitutes a window during which mitigation or control measures can be established.

## Appendices

FORTRAN90 program to study percolation in a 115X3 square grid

Program PercolacionGrilla

    implicit none

    integer::n,i,j,jj,jjj,k, muda,c(115,3)

    real::prop(100,100000),perc(100,100000),fpropc(100),fperc(100), proporcion, u, sumacum

    !Initializing

    call random_seed()

    do n=1,100000

        do i=1,115

            do j= 1,3

            c(i,j)=0

            end do

        end do

        !randomly generating cases in cells

        do i=1,100

            sumacum = 0

            do jj=1,115

                do jjj = 1,3

                    call random_number(u)

                    if (u < 0.01*i) then

                    c(jj,jjj)=1

                    else

                        c(jj,jjj)=0

                        end if

                        sumacum=sumacum+c(jj,jjj)

                end do

                proporcion = sumacum/345

            end do

            prop(i,n)=proporcion

            !looking for a free way (percolation)

            do k =1,114

           If (((c(k, 1) .eq. 1).and.(c(k + 1, 1) .eq. 1)).or.((c(k, 1).eq. 1).and.(c(k + 1, 2).eq. 1)).or.&

           ((c(k, 2).eq.1).and.(c(k + 1, 1).eq.1)).or.((c(k, 2).eq.1).and.(c(k + 1, 2).eq.1)) .or.&

           ((c(k, 2).eq.1).and.(c(k + 1, 3).eq.1)).or.((c(k, 3).eq. 1).and.(c(k + 1, 2).eq.1)).or.&

           ((c(k, 3).eq.1).and.(c(k + 1, 3).eq.1))) then

                muda = 0

                 else

                    Go To 100

                end if

                end do

            perc(i,n) = 1

            go to 200

            100 perc(i,n)=0

            200 print *,n,i, prop(i, n), perc(i, n)

        end do

        end do

    !calculating average proportions and wrintng in a file PG.TXT

    open (13, file="C:/Users/Usuario/Desktop/ESTADISTICA/FORTRAN/PG.txt")

    do i=1,100

        fpropc(i)=0

        fperc(i)=0

        do n = 1,100000

        fpropc(i)=fpropc(i) + prop(i, n)

        fperc(i)= fperc(i) + perc(i, n)

        end do

            fpropc(i) = fpropc(i) / 100000.0

            fperc(i) = fperc(i) / 100000.0

            write(13,*)fpropc(i), fperc(i)

    end do

    close(13)

end program PercolacionGrilla
